# Monovalent ions and stress-induced senescence in human mesenchymal endometrial stem/stromal cells

**DOI:** 10.1038/s41598-022-15490-2

**Published:** 2022-07-01

**Authors:** Alla Shatrova, Elena Burova, Natalja Pugovkina, Alisa Domnina, Nikolaj Nikolsky, Irina Marakhova

**Affiliations:** Department of Intracellular Signaling and Transport, Institute of Cytology, Russian Academy of Sciences, Tikhoretsky Ave. 4, St-Petersburg, Russia 194064

**Keywords:** Senescence, Permeation and transport, Apoptosis, Cell biology, Stem cells

## Abstract

Monovalent ions are involved in growth, proliferation, differentiation of cells as well as in their death. This work concerns the ion homeostasis during senescence induction in human mesenchymal endometrium stem/stromal cells (hMESCs): hMESCs subjected to oxidative stress (sublethal pulse of H_2_O_2_) enter the premature senescence accompanied by persistent DNA damage, irreversible cell cycle arrest, increased expression of the cell cycle inhibitors (p53, p21) cell hypertrophy, enhanced β-galactosidase activity. Using flame photometry to estimate K^+^, Na^+^ content and Rb^+^ (K^+^) fluxes we found that during the senescence development in stress-induced hMESCs, Na^+^/K^+^pump-mediated K^+^ fluxes are enhanced due to the increased Na^+^ content in senescent cells, while ouabain-resistant K^+^ fluxes remain unchanged. Senescence progression is accompanied by a peculiar decrease in the K^+^ content in cells from 800–900 to 500–600 µmol/g. Since cardiac glycosides are offered as selective agents for eliminating senescent cells, we investigated the effect of ouabain on ion homeostasis and viability of hMESCs and found that in both proliferating and senescent hMESCs, ouabain (1 nM–1 µM) inhibited pump-mediated K^+^ transport (ID_50_ 5 × 10^–8^ M), decreased cell K^+^/Na^+^ ratio to 0.1–0.2, however did not induce apoptosis. Comparison of the effect of ouabain on hMESCs with the literature data on the selective cytotoxic effect of cardiac glycosides on senescent or cancer cells suggests the ion pump blockade and intracellular K^+^ depletion should be synergized with target apoptotic signal to induce the cell death.

## Introduction

Monovalent ions are involved in the control of cell growth, proliferation, and death. Unlike Ca^2+^, which is an important signaling player in the cell, the role of monovalent ions such as K^+^, Na^+^ and Cl^−^, in these cellular processes is not well understood. It is commonly suggested that ion transporters and ion channels are involved in the intracellular signaling network, and K^+^, Na^+^, and Cl^−^ are important for setting the membrane potential and the intracellular pH and Ca^2+^ concentrations during cell cycle progression. For example, the concentration of Na^+^ in cells can affect cell cycle progression by pH_i_ changes: it has been shown that Na^+^/H^+^ exchanger activity regulates G_2_/M progression by increasing pH_i_ which, in turn, affects cyclin B1 expression and cdk2 activity^1–3^. It is assumed that cellular Cl^−^ is involved in the hyperpolarization of cell membrane during G_1_/S transition^4^.

In addition to signaling role, the transmembrane movement of monovalent ions may be important in context of cell volume control. Cell division depends on cell volume increase, and monovalent ions (such as K^+^, Na^+^, Cl^−^) play important role in the regulation of cell volume^5–8^. Cell volume is considered as an important component in the regulation of cell cycle progression. In studies of transformed cells of different origin and human mesenchymal endometrial stem cells we revealed significant changes in cell K^+^ content and K^+^ influxes which were related to cell accumulation in G_1_ phase of cell cycle and proliferation slowing^9–11^. Analysis of K^+^ content changes in cell cultures with different proliferative status and in human blood lymphocytes, stimulated to growth, suggested that K^+^ as the main intracellular ion might be involved in regulation of cellular water content during cell transit from quiescence to proliferation^12,13^. On the whole, being elements of cellular “housekeeping”, main monovalent permeable ions are able to modulate intracellular signaling and provide a specific intracellular ion context for development of peculiar cellular response.

In recent years, ion channels and transporters, Na^+^, K^+^-ATPase in particular, are suggested as anti-aging targets. It has been revealed that cardiac glycosides selectively kill senescent cells via apoptosis^14,15^. It is proposed that cardiac glycosides damage harmful cells by blocking Na^+^, K^+^-ATPase, however the mechanism underlying their selective killing effect has not been established.

Cellular senescence is defined as an irreversible cell cycle arrest that can be triggered in cells in response to various intrinsic and extrinsic stimuli, as well as developmental signals^16–20^. Senescence plays physiological role during normal cell development, it underlies stem cell aging and is also proposed as a tumor suppression mechanism^21–23^. Senescence markers such as DNA damage, increased expression of the cell cycle inhibitors (p53, p21) as well as distinct phenotypic alterations, including chromatin remodeling, metabolic reprogramming, characteristic messaging secretome can be used to identify senescent cells. It is generally accepted that senescence is a very heterogeneous phenomenon, and markers proposed for identifying senescence are neither specific nor universal. For example, they do not always make it possible to distinguish senescence from quiescence or reversible arrest of the cell cycle^24,25^.

Despite profound metabolic changes and impaired protein synthesis, alterations in mitochondria and lysosomes physiology, senescent cells remain metabolically active for a long time. Whether monovalent ions participate in senescence development as well as in maintaining the viability of senescent cells has not been investigated. To date, there are few studies on changes in ion content during senescence. Higher concentrations of intracellular Ca^2+^ and activation of Na^+^/H^+^ exchange are commonly observed in senescent cells compared to proliferating cells^3,26–28^. The membrane potential of both cells and mitochondria appears to be lower in aging cells than in cyclic cells^14^. Using fluorescent probes, an increased content of K^+^ and Na^+^ was detected in the senescent human lung fibroblast IMR90^15^. Direct analysis of K^+^ and Na^+^ in senescent cells has not yet been performed.

In this study, we investigated the homeostasis of monovalent ions during the premature senescence development. Using flame photometry to assess cellular K^+^ and Na^+^ content and transmembrane Rb^+^ (K^+^) fluxes, we studied changes in ion transport during oxidative stress-induced senescence in human mesenchymal stem cells. In the adult body, these cells exist in various tissues and are responsible for the replenishment of cells and the regeneration of damaged tissues. In our investigation, we used human endometrial mesenchymal stem/stromal cells (hEMSCs) derived from menstrual blood^29^. Isolated from endometrium these cells represent a stromal population containing a subpopulation of stem cells, progenitors and differentiated cells a heterogeneous population that adheres to plastics and consists mainly of endometrial glandular and stromal cells. The easy and non-invasive extraction of hEMSCs from menstrual blood, their multipotency and high proliferative activity in vitro without karyotypic abnormalities demonstrate the potential of using these cells in regenerative medicine.

## Results

### H_2_O_2_—stressed hMESCs enter premature senescence

**Figure 1:**
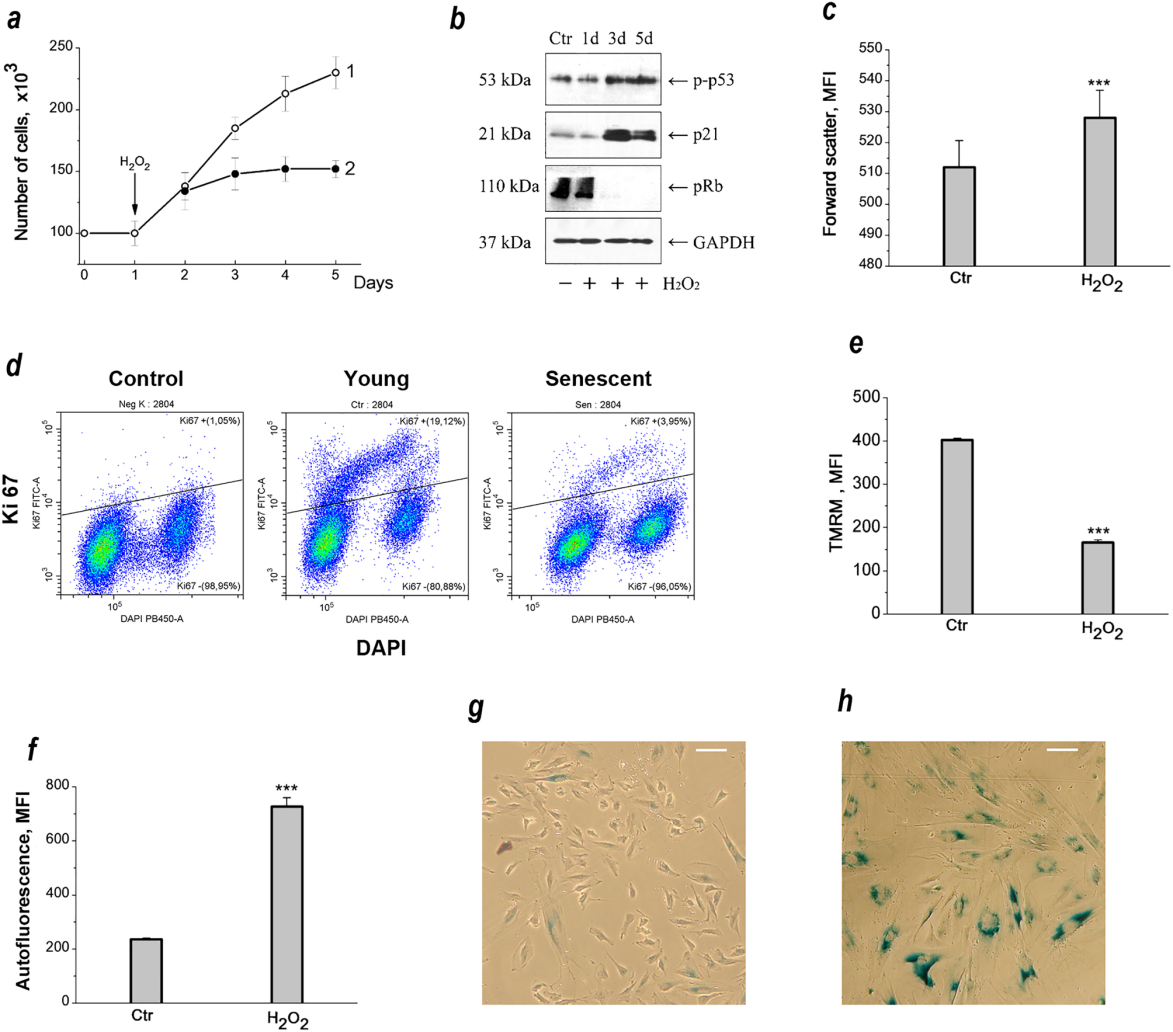
Oxidative stress induces premature senescence in hMESCs. (**a**) Growth curves of control (1) and H_2_O_2_-treated (2) hMESCs. (**b**) H_2_O_2_-induced activation of p53/p21/Rb pathway in hMESCs. Western blot analysis of p53 and Rb phosphorylation levels as well as p21 protein expression performed at indicated time points after H_2_O_2_ treatment. Representative results of the three experiments are shown. Ctr—control (untreated) hMESCs cultured in standard conditions; GAPDH was used as loading control. The original blots were cut prior to antibody treatment during blotting. The original blots are provided in Supplementary Fig. 1b. (**c**) Forward scatter reflecting cell size change on the 5th day after the oxidative stress. (**d**) Cell cycle phase distribution of the proliferation marker Ki-67 in proliferating young and senescent hMSCs. FITC mouse IgG1 is presented as a negative control. Representative Pseudo Color Plots are shown. (**e**) Mitochondrial membrane potential is decreased on the 5th day after oxidative stress as revealed by TMRM (tetramethylrhodamine, methyl ester) fluorescence. (**f**) Lipofuscin accumulation estimated by autofluorescence measurement on the 5th days after the stress. (**g, h**) Representative microphotographs of SA-β-Gal staining in control (**g**) and stressed hMESCs (**h**). Scale bar is 50 µm. In (**a**), (**c**), (**e**) and (**f**) data are presented as mean ± SD (n = 3), ***p < 0.005 versus the control cells (Ctr). MFI, mean fluorescence intensity; SA-β-Gal, senescence associated β-galactosidase.

It has recently been found that hMESCs subjected to sublethal oxidative stress undergo an irreversible cell cycle arrest mediated by the p53/p21/Rb pathway and exhibited a senescent phenotype including persistent DNA damage foci, cell hypertrophy, enhanced β-galactosidase staining^30^. Herein, we applied this cell model to study the ion homeostasis during the stress-induced premature senescence. As seen in Fig. 1a, the time course of the growth curves indicates gradual growth arrest of cells treated with sublethal pulse of H_2_O_2_ (200 µM, 1 h) compared to proliferating control cells. Stress-induced cell cycle arrest is mediated by the p53/p21/Rb pathway. As shown in Fig. 1b, on the 3rd day after H_2_O_2_-treatment, the levels of both p53 phosphorylation and p21 expression significantly increased. Of note, the observed upregulation of p53 and p21 persisted 5 days after senescence induction, suggesting a permanent cell cycle arrest typical of senescence. In addition a proliferative status of cells was examined by staining with antibodies against Ki67, a marker of cycling cells. There was a significant decrease in the number of Ki67-positive cells in stressed hMESCs population, while the control young cells had a pronounced staining for Ki67 (Fig. 1d).

The H_2_O_2_-stressed hMESCs cultures also showed a number of senescent-associated biomarkers. As evidenced by forward scattering analysis, sublethal H_2_O_2_ induced an increase of cell size after 5 days (Fig. 1c). Stress-induced arrested hMESCs displayed the decreased mitochondrial membrane potential as evaluated by decreased TMRM fluorescence signal^31,32^ (Fig. 1e). Also, the enhanced autofluorescence of stressed cells indicated accumulation of lipofuscin, which is considered as a marker of cellular senescence^33,34^ (Fig. 1f). Finally, the SA-β-galactosidase activity, a commonly used marker of senescence, was increased in arrested hMESCs (Fig. 1g,h). Of importance, H_2_O_2_ pretreated senescent hMESCs retain high viability. As evaluated by FACS analysis, at the 7th day the percentage of viable cells in stressed population was 91 ± 7 (n = 3) instead of 96 ± 5 (n = 3) in control proliferating cells. Taken together, these data suggest that hMESCs subjected to a sublethal oxidative stress represent an adequate model for studying ion homeostasis during premature senescence progression.

### Cell K^+^ and Na^+^ content during senescence progression of stressed hMESCs

**Figure 2:**
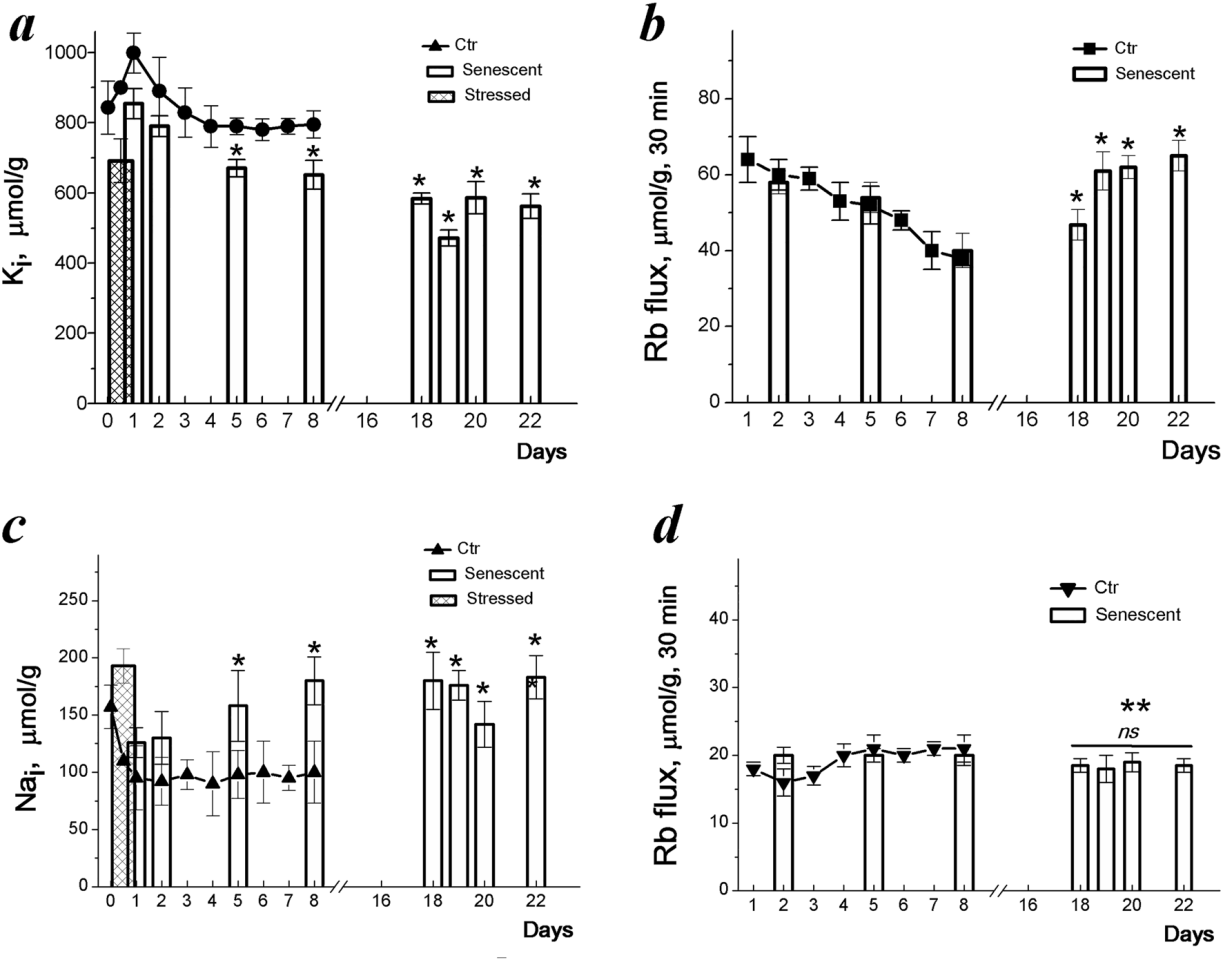
Intracellular K^+^ and Na^+^ content and Rb^+^ influxes during senescence development in stress-induced hMESCs. Ion content in young (Ctr) and senescent hMESCs were assayed during the first 8 days of cultivation and then, in late senescent cells between 18th and 22nd days. (**a**, **c**) Cell K^+^ (**a**, *circles*) and Na^+^ (**c**, *triangles up)* content in young hMESCs during culture growth and in stressed hMESCs during senescence development (*bar graphs*). *Dashed bar graphs* represent cation content just after treatment of cells with 200 μM H_2_O_2_ for 1 h. (**b**, **d**) Ouabain-sensitive (**b,** *squares*) and ouabain-resistant (**d**, *triangles down***)** Rb^+^ influxes in cycling hMESCs during culture growth and in stressed hMESCs during senescence development (*bar graphs*). Data presented as means ± SD of six (the first 8 days) and of three (18–22 days) independent experiments. Significant difference was calculated using one-way ANOVA-Tukey tests for young (Ctr) and senescent cells, *p < 0.05; *ns,* not significant, **p < 0.001.

Short oxidative treatment (200 μM H_2_O_2_ for 1 h) leads to a decrease in K^+^ content and an increase in the Na^+^ content in proliferating hMESCs (Fig. 2a). As a result of reciprocal changes in the content of K^+^ and Na^+^ in H_2_O_2_-treated cells, the intracellular K^+^/Na^+^ ratio decreased from 7–8 to 3–3.5 which indicates disordered ionic gradients during oxidative stress. After replacing the medium with a fresh medium that did not contain H_2_O_2,_ the ionic gradients were restored within a day, however, the K^+^ content in stressed cells was found to be lower than in control proliferating cells (Fig. 2a).

We then assessed the cellular monovalent ions during culturing stressed hMESCs. In our experiments, during the first 2 days, the stressed cultures continued to grow, though at a slower rate than the control cultures, and then stopped growing (Fig. 1a). Under these conditions, in stressed cultures, the content of K^+^ decreases similarly to how it decreases in proliferating normal cells, which is associated with an increase in cell density and a concomitant decrease in the proliferative activity of the culture^11^. By the 8th day, the K^+^ content decreases to a constant level, but in stressed cells it is lower (652 ± 41 μmol/g) than in control cyclic cells (795 ± 39 μmol/g) (Fig. 2a). Thus, stress-induced cessation of cell proliferation is accompanied by a decrease in the K^+^ content calculated for the protein content in the cell.

To assess the content of cations in a cell, in our studies the measured amount of cations was normalized to the mass of cellular protein in the same sample. In cell biology, such evaluation of intracellular ions is widely used. Indeed, there are significant difficulties in assessing intracellular ion concentrations (ion content per cell water content) because of the difficulties in measuring the volume and water in adherent cells. The most adequate method for assessing the water content of cells in suspension cultures—measuring the buoyant cell density—is not applicable for monolayer cell cultures. It should be noted here that there are attempts to assess the ionic and other physiological parameters of cells in monolayer cultures after their treatment with a trypsin-containing medium. Our experience has shown, that cells shortly treated with trypsin (0.05%) have an increased Na^+^ content (while maintaining a high K^+^ content) and a K^+^/Na^+^ ratio close 1 (Table 1). It also turned out that a low K^+^/Na^+^ ratio persists if the cells are washed from trypsin in a fresh medium and then kept in suspension for up to 1–2 h (longer observations were not carried out). It is noteworthy that only after attachment to the adhesive surface the low Na^+^ content and the high K^+^/Na^+^ ratio are restored in these cells (Table 1). Based on these data, we believe that the flame emission method used to measure the intracellular ion content is the most appropriate for studying the ionic homeostasis of monolayer cultures. The method allows one to determine both the content of basic cations in cells and the ion influxes, using analogous cations (for example, Rb^+^ to assess the influx of K^+^). It is also important that normalization of the amount of ions (in our case, K^+^) to the amount of protein in each sample allows us to obtain data that contribute to understanding the mechanism of K^+^ participation in cell growth and proliferation.

**Table 1:** Cell K (K_i_) and Na (Na_i_) content in hMSECs treated with tripsin-containg medium and seeded into new culture.

		Cation content µmol/g		K_i_/Na_i_
		K_i_	Na_i_	
1	Control cells in monolayer confluent culture			
		631 ± 21	90 ± 3	7.01
2	Cells were detached with 0.05% trypsin-containing medium, washed and suspended in fresh culture medium			
	**Time in suspension**			
	2 min	624 ± 28	663 ± 23	0.94
	30 min	626 ± 20	586 ± 32	1.08
	1 h	630 ± 18	590 ± 25	1.08
3	Trypsin-treated, suspended cells are seeded in a new monolayer culture			
	**Time after seeding**			
	1 h (attached cells)	644 ± 40	108 ± 9	5.03
	24 h (proliferating cells)	810 ± 24	118 ± 18	6.86

Control cells (1) were from confluent proliferating culture of hMSECs. Cellular K and Na was measured by emission flame photometry as described in Methods.

Senescent hMESCs remain viable in culture for a long time. We asked if the late stressed cells retain a high K^+^/Na^+^ ratio. As seen in Fig. 2a, in long-term cultures (up to 22 days), senescent hMESCs had a K^+^ content (562 ± 39 µmol/g), comparable to that in early senescent cells. After oxidative stress, the content of intracellular Na^+^ decreases and does not change for 8 days, however during senescence progression, the content of Na^+^ in cells increases from 120 ± 10 to 160 ± 19 µmol/g (Fig. 2c). Taken together, these data indicate that, during long-term culture, senescent hMESCs maintain the high K^+^/Na^+^ ratio typical for functionally active animal cells. A peculiar feature of senescent hMESCs in comparison with proliferating cells is a lower K^+^ content per cellular protein.

### Na^+^,K^+^-ATPase-mediated Rb^+^ (K^+^) transport is increased during senescence progression in stressed hMESCs

Short-term Rb^+^ uptake was used to assess changes in K^+^ transport during senescence. In proliferating hMESCs, ouabain-inhibitable Rb^+^ influx, mediated by the Na^+^,K^+^-ATPase pump, accounts for more than half of the total Rb^+^ flux. In the first days after stress, the ouabain-inhibitable Rb^+^ flux decreases, as well as in proliferating cultures, the influx decreases (Fig. 2b). A decrease in pump-mediated K^+^ transport in growing cell culture is associated with density-dependent inhibition of cell proliferation^11^. In stress-induced hMESCs, a decrease in ouabain-inhibitable Rb^+^ influx also reflects the transition to cell cycle arrest and the cessation of cell proliferation.

We then investigated the transport activity of the Na^+^, K^+^-ATPase pump in hMESCs during senescence development and compared ouabain-inhibitable Rb^+^ influxes in proliferating and early stressed hMESCs with that in late stressed hMESCs. As can be seen in Fig. 2b, in late stressed cells, the ouabain-inhibitable Rb^+^ uptake was increased accounting 65 ± 4 µmol/g, 30 min (n = 7) instead of 40 ± 4 µmol/g, 30 min (n = 6) in early stressed cells. These data indicate elevated pump-mediated K^+^ transport in established senescent hMESCs. To determine whether the observed increase in pump activity is proportional to changes in intracellular Na^+^ concentration, we compared pumping rate coefficients calculated as the ratio of ouabain-inhibitable Rb^+^ uptake to intracellular Na^+^ content during senescence development^35–37^. It turned out that the rate coefficients do not differ for early and late stressed cells. Thus, the increased ouabain-inhibitable K^+^ transport in senescent cells is not associated with a change in the intrinsic properties of Na^+^/K^+^-ATPase pump, but is a consequence of flux-concentration relations in existing ion pumps and is probably associated with an increase in cellular Na^+^ in late senescent hMESCs.

During senescence development, passive transport of Rb^+^(K^+^), resistant to ouabain, decreases slightly and then remains unchanged (Fig. 2d).

Comparison of ion changes during the growth of hMESC culture with those in senescent cells shows that stress-induced cell cycle arrest and senescence progression are accompanied by a decrease in K^+^ content per gram of cellular protein mass. The senescence progression is also associated with elevated cell Na^+^ content and increased pump-mediated K^+^ influxes.

### Cell K^+^ and Na^+^ content and Rb^+^ fluxes in ouabain-treated cycling and senescent hMESCs

Having data on ion homeostasis of hMSCs, both proliferating and senescent, we analyzed the cytotoxic effect of ouabain on these cells. Increasing evidence suggests that cardiac glycosides are capable of inducing apoptosis and selectively killing senescent, but not cycling cells^14,15^. We asked whether ouabain-induced changes in cellular K^+^ and Na^+^ content could contribute to apoptotic cell death in senescence, and examined the relations between changes in ionic homeostasis in the presence of ouabain and ouabain’s ability to kill senescent hMESCs. So far, direct measurements of intracellular K^+^ and Na^+^ content have not been performed with ouabain-treated senescent cells compared with cycling cells.

**Figure 3:**
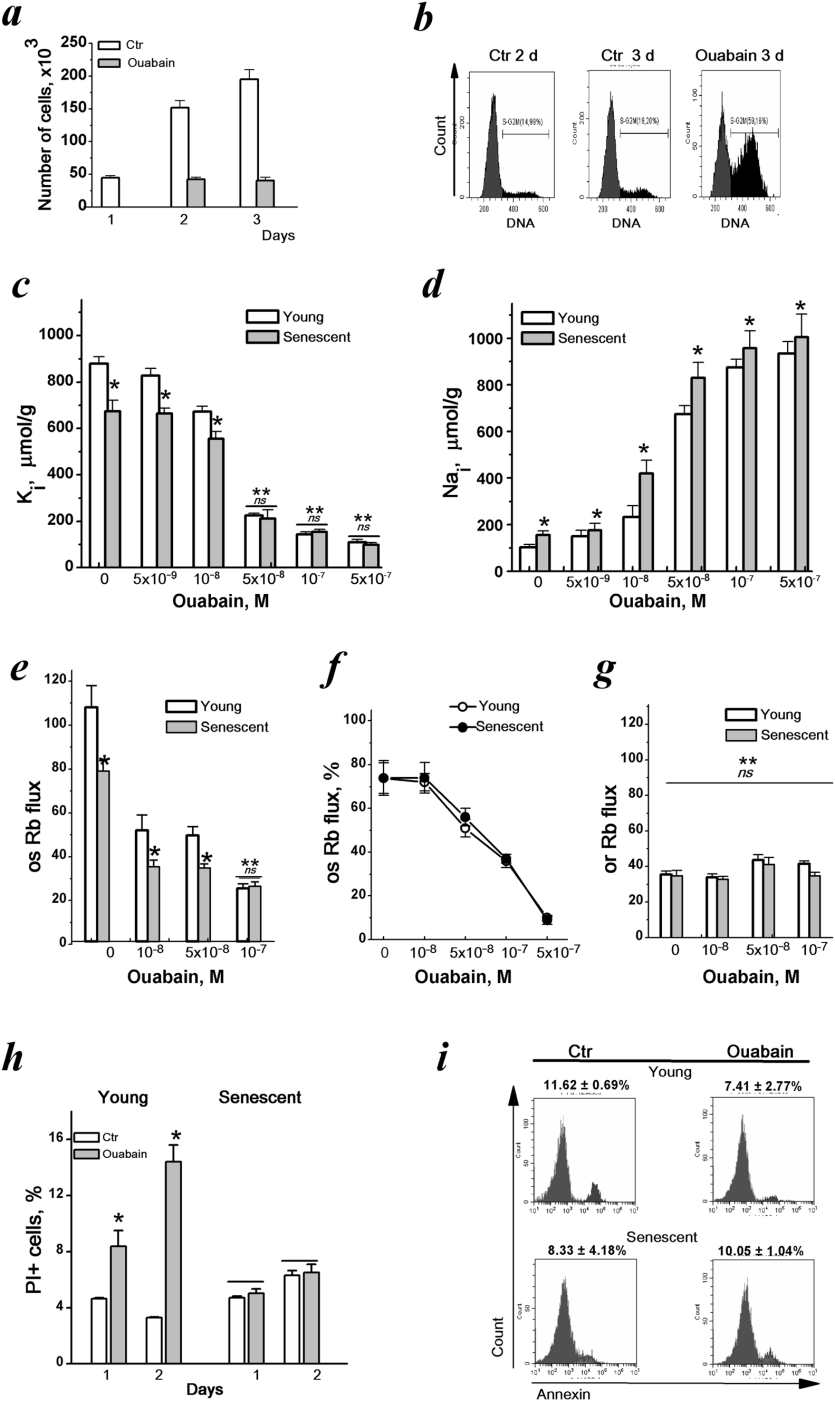
Ouabain treatment lead to severe changes in cell K^+^ and Na^+^ content and Na^+^/K^+^ pump-mediated transport, but did not induce apoptosis in young and senescent hMESCs. (**a**, **b**) Ouabain (5 × 10^-7^ M) inhibits cell growth (**a**) and induces G_2_/M block in hMESCs (**b**). (**c**, **d**) Changes of cell K^+^ (**c**) and Na^+^ (**d**) content in young (*open bars*) and senescent (*filled bars*) hMESCs. (**e–g**) Ouabain inhibits pump-mediated (os) Rb^+^ influx in concentration-dependent manner in young and senescent hMESCs (**e**, **f**) but did not affect the passive (or) Rb^+^ influx (**g**). Rb^+^ fluxes are presented s µmol/g, 30 min. (**h**, **i**) Ouabain treatment (10^–6^ M, 2 days) increase the number of “dead” PI + cells in young hMESCs (**h**) and do not induce apoptosis both in young and senescent hMESCs (**i**). Data are shown as mean ± SD (n = 3–5). Significant difference was calculated using one-way ANOVA-Tukey tests for young and senescent cells (**c**, **d**, **e**, **g**), and for young control and ouabain-treated cells (**h**), *p < 0.05; *ns,* not significant, **p < 0.001.

First, we confirmed that ouabain at high concentrations stops cell proliferation. Starting from concentration 10^–7^ M by 24 h ouabain inhibits the growth of hMESCs cultures causing S-G_2_/M delay in cell cycle (Fig. 3a,b). As expected, after incubating with ouabain for 24 h, there occur reciprocal changes in cell K^+^ and Na^+^ contents (Fig. 3c,d). With increasing the ouabain concentration from 10^–9^ to 10^–7^ M the cell K^+^ decreased from 879 ± 30 µmol/g (n = 4) to 110 ± 12 µmol/g (n = 3) in control (young cycling) cells and from 674 ± 48 µmol/g (n = 3) to 96 ± 17 µmol/g (n = 3) in senescent cells with the most significant decrease between 5 × 10^–9^ and 10^-8^ M concentrations in both cell populations. Simultaneously with a decrease in cellular K^+^ content, the Na^+^ content in ouabain-treated cells increased reaching the highest level with 10^–7^ M ouabain (Fig. 3b*)*. Ouabain induced dose-dependent decrease in ouabain-inhibitable Rb^+^ influx with the same IC_50_ (5 × 10^–7^ M) for both young and senescent hMESCs (Fig. 3e,f). Within the wide range of ouabain concentrations, ouabain-resistant Rb^+^ influx did not change (Fig. 3g). Thus, ouabain already at a concentration of 10^–7^ M completely stops ion pumping thus leading to disruption of cell K^+^/Na^+^ gradients in both young and senescent hMESCs.

We then tested how long-term ouabain affects the viability of young and senescent hMESCs. According to FASC analysis, 10^–6^ M ouabain resulted in an increase in the number of PI-stained young proliferating cells (8.66%PI + and 14.43%PI + after 1 and 2 days) whereas in the population of ouabain-treated senescent hMESCs the number of PI-stained cells remained as low as in the control (5.02%PI + and 6.07%PI +) (Fig. 3h). As shown by Annexin V test (Fig. 3i), 10^–6^ M ouabain did not lead to apoptosis induction both in young (11.62%AnV + in control and 7.41%AnV + in the presence of ouabain) and in senescent hMESCs (8.83%AnV + in control and 10.05%AnV + in the presence of ouabain). Taken together, these data indicate that in young cycling and senescent arrested hMESCs, ouabain causes the same disturbances in monovalent ion homeostasis and does not induce apoptosis.

## Discussion

In the present study, we investigated ion homeostasis during stress-induced cell cycle arrest and premature senescence development in hMESCs. Using flame photometry to estimate cellular K^+^ and Na^+^ content and transmembrane Rb^+^ (K^+^) fluxes we found that senescent hMESCs maintain high K^+^/Na^+^ ratio typical for functionally active animal cells. The senescence progression is accompanied by elevated Na^+^ content in cells and increased pump-mediated K^+^ influxes. Stress-induced cell cycle arrest does not affect passive, ouabain-resistant K^+^ fluxes across plasma membrane. A peculiar feature of senescent in comparison with proliferating hMESCs is lower K^+^ content per cell protein mass.

Premature senescence caused by various stresses in cells is associated with the cessation of cell proliferation. The lower ratio of the K^+^ content to protein mass in senescent cells is in good agreement with our previous finding that a decrease in this index correlates with a decrease in cell proliferation^9–11^. Recently, when studying the activation of human T lymphocytes, we also found that the transition of cells from quiescence to proliferation is necessarily accompanied by an increase in the content of both K^+^ and water per g of protein so that during the growth of lymphocytes and an increase in their volume, the intracellular concentration of K^+^ remains constant^13^. These data allow us to suggest that K^+^ may be involved in maintaining cell growth and proliferation as an intracellular ion, which participates in the regulation of cell volume by adjusting the water balance of cell.

In the present study, by analogy with our previous experimental data when reliable measurements of K^+^, water and volume were carried out simultaneously on proliferating and resting cells in suspension^13,38,39^, relying on a theoretical analysis of ion and water balance in animal cells^5,35^, and also taking into account that K^+^ is the main cation compensating intracellular anions we assume that the lower ratio of cell K^+^ content to cell protein mass can be interpreted as lower water content in senescent hMESCs. To find out if this is really so, it is necessary to take reliable measurements of the volume of senescent cells. Various experimental approaches to measuring cell volume have been developed; however, this is still not an easy task to measure the volume of adherent cells in growing monolayer cultures^40^.

There are few experimental data on changes in cell hydration state associated with a change in the proliferative status of cells. Based on some evidence of higher embryonic and cancer cell hydration, increased cell hydration has been proposed as an important factor in cell malignant growth and carcinogenesis^41^. The relationship between hydration and cell proliferation may reflect the effect of macromolecular crowding on cellular metabolism: macromolecular crowding reduces metabolic processes and cytoplasm fluidity^42–45^. It has also been hypothesized that macromolecular crowding plays a role in signaling volume perturbations^6,46–50^. Finally, measurements of water content in aging erythrocytes have suggested that cell water loss and macromolecular crowding may be a common mechanism of cellular senescence^51^.

To further investigate the role of monovalent ions in the maintenance of senescence, we decided to test the participation of the Na^+^/K^+^ pump in the survival of senescent hMESCs. This problem is discussed in view of proposed anti-aging effects of cardiac glycosides: cardiac glycosides have been reported to selectively kill senescent cells via apoptosis and can be used as senolytics in the treatment of aging-related diseases^14,15^. In our experiments, in proliferating and senescent hMESCs, ouabain inhibits pump-mediated K^+^ transport in a dose-dependent manner, which leads to profound changes in ionic homeostasis, such that Na^+^ substitutes K^+^ inside the cell and after a 24 h incubation with 0.1 µM ouabain, in cells of both types, intracellular K^+^ becomes only 90–100 instead of 800–900 µmol/g protein in untreated cells. Despite such profound disturbances in ion homeostasis, as assessed by PI/Annexin staining, long-term ouabain (1 µM, up to 48 h) did not induce death and apoptosis in senescent hMESCs.

Earlier it was reported about high apoptosis resistance of human mesenchymal stem cells^30,52–54^. However, the high resistance of senescent hMESCs to ouabain is not consistent with proposed selective cytotoxic (senolytic) activity of cardiac glycosides in relation to senescence^14,15^. Note that there is a lot of data in the literature that cardiac glycosides also kill high proliferating cancer cells, but not cells that normally cycle^55–59^. The question arises as to what may underlie the selective toxic effect of cardiac glycosides on senescent or cancer cells, and whether disturbances in cell ion homeostasis (in particular, K^+^ content) can be involved in the anti-aging or anticancer effects of these drugs.

The Na^+^/K^+^-ATPase pump, a cellular target of cardiac glycosides, is a major player in glycoside cytotoxic effects. Depletion of Na^+^/K^+^-ATPase by RNA interference inhibited glycoside-induced apoptosis in cells^60^. Several studies correlate the expression levels of the alpha-1 Na^+^, K^+^-ATPase to the susceptibility of cells towards cardiac glycosides. Indeed, the expression level of Na^+^, K^+^-ATPase is dependent on cell proliferative status: quiescence is characterized by a lower expression of α1- and β1-Na^+^, K^+^-ATPase subunits, while the transition of cells to proliferation is accompanied by increasing their transcription and the synthesis of new α1-Na^+^, K^+^-ATPases^61,62^. As a result, highly proliferating cancer cells are characterized by a higher Na^+^, K^+^-ATPase expression and may be more sensitive to glycosides than quiescent, differentiated or normally cycling cells. As for senescent cells, there are no studies indicating an increased expression of Na^+^,K^+^-ATPase during senescence. In the present study, no differences were found in the relationship between K^+^ influx and ouabain dose, or in the rate coefficient for the Na^+^, K^+^-ATPase pump in proliferating and senescent hMESCs. Thus, it is unlikely that the Na^+^, K^+^-ATPase pump in senescent cells is more sensitive to cardiac glycosides. In addition, the glycoside concentrations which are toxic to senescent cells but do not to normal cycling cells fall into a narrow window^14^. These features of drugs indicate a rather low toxic specificity of glycosides in relation to senescent cells.

The Na^+^, K^+^-ATPase pump is the primary ion transport system responsible for the maintaining the Na^+^ and K^+^ concentrations and the resting membrane potential in the cell^63–66^. Animal cell stability and survival depend on the operation of the Na^+^/K^+^ pump through the pump-leak mechanism^5^. Impermeant anionic molecules in cells establish an unstable osmotic condition (the Donnan effect), which is counteracted by the operation of Na^+^/K^+^ pump, thus creating an asymmetric distribution of Na^+^ and K^+^ and preventing excessive water influx. In most animal cells, the Na^+^/K^+^ pump blockade leads to cell swollen, membrane disruption and necrotic death. To understand the mechanism of selective toxic action of glycosides on dangerous cancer or senescent cells, it is important to know what type of death they cause.

Accumulating evidence indicate that in senescent and cancer cells, cardiac glycosides induce apoptosis or “mixed” cell death with signs of apoptosis and necrosis or autophagy, causing mitochondria dysfunction^15,67–72^. In this context, K^+^ transport plays a central role in mitochondrial physiology. Under normal physiological conditions, the high electric potential difference generated by the proton pump across the inner mitochondrial membrane is used to make ATP and determines also the inward K^+^ flux and the concomitant water influx into matrix, which are compensated by the electroneutral K^+^/H^+^ antiporter (so called K^+^ cycle)^73^. Disruption of K^+^ cycle inevitably leads to disturbances in mitochondria function and contribute to intrinsic apoptosis induction. It is important, that the activities of K^+^/H^+^ antiporter and K^+^ channels are regulated by the apoptotic Bcl-2 proteins^74–76^.

The question is what might be the role of cellular K^+^ depletion due to ion pump inhibition in mitochondria dysfunction. Excessive K^+^ efflux and intracellular K^+^ loss are key early step in apoptosis induction^7,77–81^. It is noteworthy that this early loss of K^+^ occurs simultaneously with the loss of water by cells and does not lead to a decrease in the concentration of K^+^ in cells^38^. At later apoptosis, however, before the release of cytochrome c, a significant decrease in the content of K^+^ and Cl^−^ assayed by X-ray microanalysis or cryo-correlative microscopy was found in the cytoplasm and mitochondria^82–84^. Late stages of apoptosis are also associated with cell shrinkage, a decrease in water content in cells and a simultaneous decrease in the cell K^+^ concentration^85^. These data suggest that in cells with the Na^+^/K^+^ pump turned off, a sever decrease of cytoplasmic K^+^ could promote intrinsic apoptosis by disruption of the mitochondria ion and volume homeostasis.

Apoptosis as a programmed cell death is controlled by a well-orchestrated genetic program so that the cell fate is dependent on the balance between pro- and anti-apoptotic members of Bcl-2 family proteins, and the ability of cardiac glycosides to induce apoptosis depends on whether the pro-apoptotic proteins are expressed in cells treated with glycoside. It has been revealed that in cancer cells, cardiac glycosides induce apoptosis by down-regulating the anti-apoptotic proteins Bcl-XL and Bcl-2 as well as Mcl-1 and increasing the pro-apoptotic proteins Bid and Bax^57,58,86–89^. In senescent human lung fibroblast IMR90 cells, cytotoxic ouabain increased the pro-apoptotic NOXA protein^15^. Based on these studies, we conclude that if a cell is proned to apoptosis (i.e. pro-apoptotic proteins are in proper functional position in cell) cardiac glycosides can contribute the apoptosis.

Cardiac glycosides do induce apoptotic death in human embryonic stem cells (hESCs) but not in human bone marrow mesenchymal stem cells (hBMMSCs) and hESC-derived mesenchymal stem cells^54^. As reported recently and also shown in our study, cardiac glycosides do not induce apoptosis in both cycling hMESCs and their senescent partners^90^. Obviously, the high resistance of hMESCs to cardiac glycosides is provided by a highly expressed anti-apoptotic program in these cells^30,52,53,91,92^. Taken together, the above data suggest that cellular K^+^ depletion is a necessary event in cardiac glycoside-induced apoptosis; however, a separate apoptotic signal is required to kill the cell, which acts together with low cellular K^+^.

In summary, oxidative stress-induced senescence in hMESCs is associated with specific changes in cell ion homeostasis, which primarily concerns intracellular K^+^. In senescent cells, pump-mediated K^+^ transport is enhanced due to the increased Na^+^ content, while passive ouabain-resistant K^+^ influxes remain unchanged. In the course of senescence development, cellular K^+^ content decreases and in established senescent cells, the ratio of K^+^ content to cellular protein becomes lower that of in cycling cells which may indicate a decrease in hydration of senescent cells. Evaluating the cytotoxic selectivity of cardiac glycosides toward senescene, we conclude that K^+^ being a key ion in mitochondria physiology is involved in intrinsic apoptotic events in cells treated with cardiac glycosides. However, to kill senescent cells, the ion pump blockade and intracellular K^+^ depletion should be synergized with the target apoptotic signal. Given the pleiotropic effects of cardiac glycosides, their use as selective drugs for eliminating the dangerous cells warrants further study: cardiac glycosides as inhibitors of the ion pump that ensures the stability and survival of all animal cells are able to kill not only harmful tumorous or senescent cells but also functionally important differentiated cells.

## Methods

### Cells and experiment design

The procedures involved human cells were performed in accordance with the standards of the Declaration of Helsinki (1989) and approved by the Institute of Cytology Ethics Committee. Written informed consent was obtained from all patients who provided tissue.

All the experiments have been performed on human mesenchymal endometrial stem/stromal cells (hMESCs) established from a desquamated endometrium of menstrual blood from healthy donors^28^. These cells exhibited properties typical of mesenchymal stem cells. The established hMESCs have fibroblast-like morphology, express standard surface markers such as CD13, CD29, CD44, CD73, CD90, CD146, CD105 and are negative for the hematopoietic markers CD19, CD34, CD45, CD117, CD130, and HLA-DR (class II); they have the ability to differentiate into adipocytes, chondrocytes and osteoblasts^28^. Besides, the isolated hMESCs partially (over 50%) express the pluripotency marker SSEA-4 but do not express Oct-4. These cells are characterized by high rate of cell proliferation (doubling time 22–23 h).

Cells were maintained in Dulbecco's Modified Eagle Medium (DMEM)/F12 (Gibco) supplemented with 10% fetal calf serum (HyClone), 1% penicillin–streptomycin (Gibco BRL, MD, USA) and 1% glutamax (Gibco BRL, MD, USA) and subcultured at 1:3–1:4 ratio twice a week. For experiments, cells were harvested by trypsinization and plated at a density of 15 × 10^3^ cells per cm^2^.

To induce premature senescence, the subconfluent cultures of hMESCs were treated with 200 μM H_2_O_2_ (Sigma-Aldrich, St. Louis, MO, USA) for 1 h, then washed twice with serum-free medium to remove H_2_O_2_, and re-cultured in fresh complete culture medium. Cells were analyzed either immediately after H_2_O_2_ shock or at selected time points after prolonged cultivation, depending on the aim of the study. To assess the effect of ouabain on the viability of hMESCs, young proliferating cells (passage 10) and cells whose senescence was induced by peroxide after an additional 5 days of cultivation were used.

### Analysis of cell K^+^ and Na^+^ content and K^+^ influx

Measurements of ions were performed essentially as described previously^11,12^. To estimate K^+^ influx Rb^+^ was used as the physiological analog of K^+^. RbCl (final concentration 5 mM) was introduced into the culture medium for 20 min. To evaluate the Na^+^, K^+^-ATPase K^+^ influx, prior to RbCl, 10^–4^ M ouabain (Sigma-Aldrich, USA) was added to culture medium. Then, cells were rapidly washed 5 times with ice-cold isotonic MgCl_2_ and cations were extracted with 1 mL of 1% trichloroacetic acid (TCA). TCA extracts were analyzed for Rb, K and Na by emission flame photometry on a Perkin-Elmer AA 306 spectrophotometer. TCA precipitates were dissolved in 0.1 N NaOH and analyzed for protein by Lowry procedure. Ouabain-sensitive Rb^+^ uptake was calculated as the differences between the mean values measured in samples incubated with and without ouabain. The intracellular ion content was expressed as amount of ions per amount of protein in each sample analyzed.

### FACS analysis of cell viability, cell proliferation, mitochondrial health and apoptosis

For FASC analysis, adherent cells were rinsed twice with PBS and harvested by trypsinization. Detached cells were pelleted by centrifugation and suspended in PBS. Samples were analyzed with CytoFLEX flow cytometer (Beckman Coulter, Brea, CA, USA) or CytoFLEX S flow cytometer (Beckman Coulter, Brea, CA USA).

To determine cell viability propidium iodide (PI) staining was used. PI is excluded by viable cells but can penetrate cell membranes of dead cells and intercalates into double-stranded nucleic acids. 50 μg/mL of PI was added to each sample just before analysis. At least 3000 events were usually collected as the main cell population. Triplicate counts were obtained for each procedure. Representative PI versus FSC dot plots allowed us to distinguish between PI-negative “live” cells and PI-positive “dead” cells.

For cell cycle analysis, each cell sample was suspended in 300 μL PBS/serum-free medium containing 200 μg/mL of saponin (Fluka, NY, USA), 250 μg/mL of RNase A (Sigma-Aldrich, MO, USA) and 50 μg/mL of PI, incubated from 30 to 60 min at a room temperature (in dark) and subjected to FACS analysis. Data were analyzed using CytExpert software (versions 1.2 and 2.0, Brea, California, USA). Dot plots (FSC versus PI) were generated to assess the distribution of cell cycle phases. For this, the cells were gated in accordance with the DNA content. At least 15,000 cells are collected for research. The experiments were repeated three times.

To evaluate the proliferation status of cell cultures, Ki67/DAPI staining was used. Detached cells were fixed, permeabilized using True-Nuclear™ Transcription Factor Buffer Set (BioLegend, USA), stained with FITC-Ki67 antibodies (DAKO F7268, USA) and DAPI (1 µg/mL) and then analyzed by FACS.

For detection of lipofuscin accumulation, the samples were analyzed for autofluorescence (AF, 488 nm laser). To evaluate the increase in the cell size which accompanies the senescence, forward scatter signal (FS) was monitored. Tetramethylrhodamine (TMRM; Invitrogen, Carlsbad, CA, USA) dye was used as mitochondrial membrane potential indicator (MMP)^33,34^. Healthy cells have functioning mitochondria and the bright fluorescence signal, respectively. Briefly, to prepare a 1 × staining solution (100 nM) TMRM stock solution (100 μM) was diluted in 1000 times with growth medium, which added to the cells.

Apoptosis was assayed using Annexin V/Alexa Fluor ™ 647 conjugate in accordance with the manufacturer’s instructions (Thermo Fisher Scientific, Waltham, MA, USA). Treated and untreated cells were harvested by trypsinization, washed with PBS, pelleted by centrifugation and adjusted to a concentration 1 × 10^6^ cells/mL in 1 × Annexin binding buffer (10 mM HEPES, 140 mM NaCl, 2.5 mM CaCl_2,_ pH 7.4). 1 × 10^5^ cells (100 μL of cell suspension) were stained with 5 μL of Annexin V conjugate and 2 μL of DAPI (final concentration 2 μg/mL) for 15 min in the dark at room temperature. Then, 400 μL of 1 × buffer was added to each sample, gently vortexed and analyzed by flow cytometry as soon as possible.

### Immunoblotting

Immunoblotting analysis was performed as described previously^93^. The cells were lysed in 1, 3 and 5 days after treatment with 200 µM H_2_O_2_ for 1 h. Protein content was determined by the method of Bradford. SDS–PAGE electrophoresis, transfer to nitrocellulose membrane, and immunoblotting with ECL (Thermo Scientific, CA, USA) detection were performed according to standard manufacturer’s protocols (Bio-Rad Laboratories, USA). Antibodies against the following proteins were used: phospho-p53 (Ser15) (clone 16G8) (1:700, # 9286), p21Waf1/Cip1 (clone 12D1) (1:1000, # 2947), phospho-Rb (Ser807/811) (1:1000, # 8516), glyceraldehyde-3-phosphate dehydrogenase (GAPDH, clone 14C10) (1:1000, # 2118), as well as horseradish peroxidase-conjugated goat anti-rabbit IG GAR-HRP (1:10,000, # 7074S). All antibodies were purchased from Cell Signaling, USA. Hyperfilm (CEA) was from Amersham (Sweden). Equal protein loading was confirmed by Ponceau S (Sigma-Aldrich, USA) staining. The original blots were cut prior to antibody treatment during blotting. Full size blots are provided in the Supplementary Fig. 1b.

### Senescence-associated β-galactosidase assay

Cells expressing senescence-associated β-galactosidase (SA-β-gal) were identified using a β-galactosidase staining kit (Cell Signaling Technology, Beverly, MA, USA) according to the manufacturer's recommendations. The kit allows determining the activity of β-galactosidase at pH 6.0, which is well detected in senescent cells.

### Statistical analysis

All data are presented as the mean with standard error of the mean from at least three independent experiments. Statistical significance was assessed using Student’s t-test in case of pair comparisons or ANOVA-Tukey test in case of multiple comparisons. *P < 0.05, **P < 0.001 and ***P < 0.005. The specific details of each experiment are provided in the corresponding figure legends.

## Supplementary Information

Supplementary Information.

## Data Availability

All data generated or analyzed during this study are included in this published article.
