# Supplementary material for: Monovalent ions and stress-induced senescence in human mesenchymal endometrial stem/stromal cells
Source: Sci Rep. 2022 Jul 1;12:11194. doi: 10.1038/s41598-022-15490-2 (PMC9249837; doi:10.1038/s41598-022-15490-2)
Supplement: Supplementary file 1 — Supplementary Information. [file 41598_2022_15490_MOESM1_ESM.pdf]

Monovalent ions and stress-induced senescence in human mesenchymal endometrial stem cells. Alla Shatrova, Elena Burova, Natalja Pugovkina, Alisa Domnina, Nikolaj Nikolsky and Irina Marakhova.

Supplementary Information.

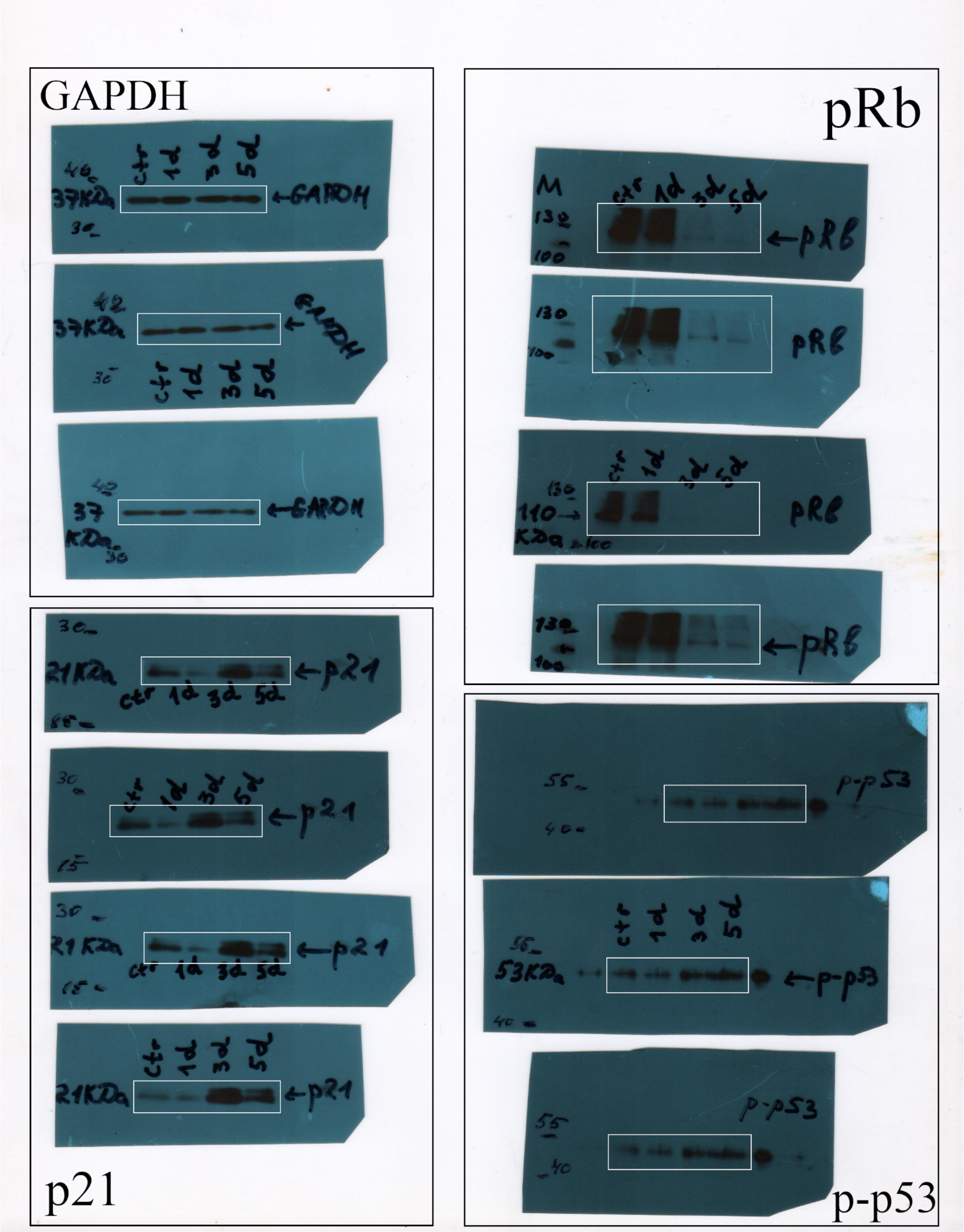

Supplementary **Figure 1b.** Original Western blots. Time-dependent alterations of functional status of p53, p21 and Rb in response to hMESC treatment with 200 μM H_2O_2, compared with control cells (Ctr). 1, 3, 5 d (days) – time points after H_2O_2 stimulation (senescence induction). GAPDH - loading control. p-p53 – phospho-p53; p21 –p21 protein; pRb – phospho-Rb. On the blot with pRb "M" presents prestained protein ladder (100kDa and 130 kDa, upper). Representative results from three independent experiments.

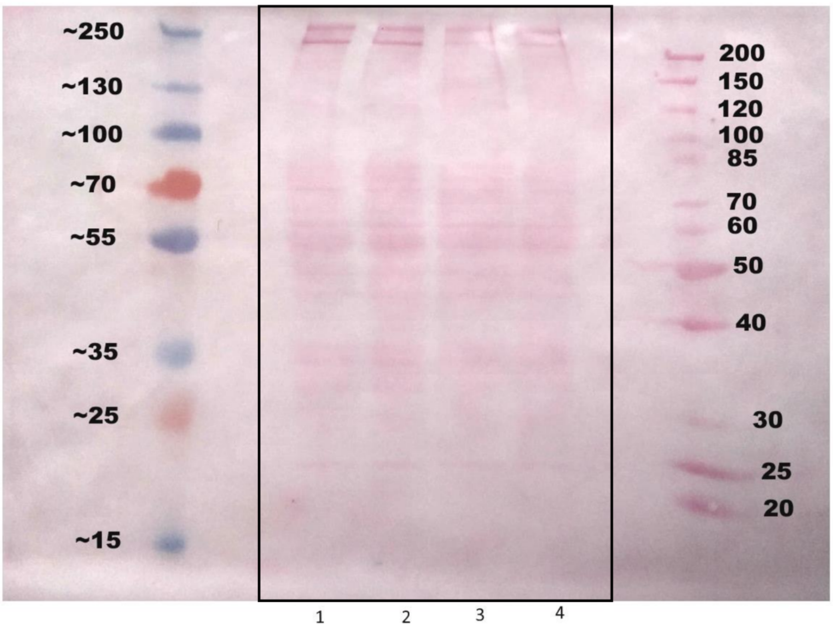

Nitrocellulose membrane after electroforetic transfer of proteins stained by Ponso S.

On the left – prestained protein ladder, on the right – unstained protein ladder. 1 – control cells, 2, 3, 4 – cells picked up in 1, 3, 5 days after H_2O_2 treatment.
